# Anthropometric characteristics between ever and never screened for hypertension in Burkina Faso

**DOI:** 10.4102/jphia.v16i1.737

**Published:** 2025-09-25

**Authors:** Jeoffray Diendéré, Toussaint Rouamba, Jean Kaboré, Augustin N. Zeba, Halidou Tinto, Sylvin Ouédraogo, Athanase Millogo, Séni Kouanda

**Affiliations:** 1 Department of Biomedical and Public Health, Research Institute for Health Sciences (IRSS), Bobo-Dioulasso, Burkina Faso; 2 Department of Biomedical and Public Health, Research Institute for Health Sciences (IRSS), Nanoro, Burkina Faso; 3 Department of Biomedical and Public Health, Research Institute for Health Sciences (IRSS), Ouagadougou, Burkina Faso; 4 Department of Medicine, Joseph Ki-Zerbo University, Ouagadougou, Burkina Faso; 5 African Institute of Public Health (IAPS), Ouagadougou, Burkina Faso

**Keywords:** screening for hypertension, blood pressure, body sizes, thin-looking build, Burkina Faso

## Abstract

**Background:**

Excess body weight was associated with a higher chance for hypertension detection.

**Aim:**

To compare the anthropometric characteristics and blood pressure levels between Burkinabè adults who had ever been screened for hypertension and those who had never been screened, and to assess the associated factors with the uptake of hypertension screening.

**Setting:**

Urban and rural Burkina Faso.

**Methods:**

This was a secondary analysis using the Burkina Faso 2013 WHO Stepwise approach to Surveillance cross-sectional survey. Data from 3831 adult men and women were analysed. Descriptive and analytical analyses were performed using Student’s *t*, ANOVA, ^χ^^2^, Fisher’s exact tests and logistic regression.

**Results:**

Among participants, 41.6% (95% CI: 40.0–43.1) had never been screened for hypertension, and compared to those who had ever been screened, they had significantly lower mean weight, waist circumference and body mass index, and lower prevalence of overweight or obesity and abdominal obesity. The prevalence of prehypertension was similar between the two groups (around 40%) and the prevalence of hypertension was lower in those who had never been screened (17.3% vs 20.8%; *p* = 0.007). Overweight or obesity (adjusted odds ratio [aOR] = 1.3; *p* = 0.03) and abdominal obesity (aOR = 1.3; *p* = 0.002) were associated with screening uptake.

**Conclusion:**

The Burkinabè adults who had never been screened for hypertension were apparently thin, but pre-hypertension or hypertension was also common among them. Increasing body size (excess weight or abdominal obesity) may be the reason for screening uptake.

**Contribution:**

Specific awareness-raising messages to motivate slim people to undergo screening need to be developed.

## Introduction

Hypertension leads to the risk for cardiovascular events, disability and premature mortality^1^, and more than four-fifths of people with hypertension live in low- and middle-income countries (LMICs).^2^ The challenge of reducing the burden of cardiovascular disease, particularly in LMICs in sub-Saharan Africa (SSA), includes efforts to improve the detection and management of hypertension.^3^ Excess body weight was a consistent determinant of hypertension in SSA^4^ and overweight or obese status has been associated with a lower risk of remaining undiagnosed for hypertension.^5^ Barriers to screening should be tracked, and it can be hypothesised that specific barriers may also be related to self-perception of body size or image in relation to susceptibility to diseases such as hypertension. The first STEPwise approach to surveillance (STEPS) survey in Burkina Faso used a nationally representative sample to assess uptake of hypertension screening and included the real-time measurements of anthropometric and blood pressure (BP) characteristics. Compared with people living with undiagnosed hypertension in Burkina Faso, people with known hypertension were more likely to be overweight (by more than 10 points difference) or obese (by more than 20 points difference).^6^

Although excess body weight is a significant risk factor for hypertension, it is also worth considering the possibility of selection bias in participation and/or screening provision. This seems to be relevant in view of the significant rate of metabolic disorders reported in Burkinabè of normal weight.^7^

Our study aimed to compare the anthropometric characteristics and blood pressure levels between adults who had ever been screened for hypertension and those who had never been screened at the time of the first STEPS survey in Burkina Faso, and to assess the factors associated with screening uptake.

## Research methods and design

### Study design

A secondary cross-sectional analysis was performed using data from the World Health Organization (WHO) STEPS^8^ survey conducted in Burkina Faso in 2013. This study is a recommended tool for surveillance of chronic diseases and their risk factors in the WHO member countries. The survey is a standardised method to collect, analyse and disseminate data. It is a sequential process that starts with gathering key information about the risk factors with a questionnaire; subsequently, simple physical measurements are collected. The WHO STEPS includes a representative sample of the study population, which allows the results to be generalisable to the entire population.

### Study population, sample size, participants’ selection and data collection

The study population was adults of both the sexes, aged between 25 and 64 years, who had been living in Burkina Faso for at least 6 months on the day of the survey.

The total sample size calculation and the data collection process throughout the country have been described in a previous publication.^9^ The calculation was based on the prevalence of hypertension as primary outcome, and was estimated at 29.6% (95% confidence interval [CI]: 27.3–31.9). The nationally-representative sample size, based on 20.0% non-response, was estimated as 4785 (rounded up to 4800) adults aged 25–64 years. Since the national adult rate of those who have ever been screened for hypertension was previously unknown, if the approximate LMICs’ rate was assumed (at about one-third),^10^ the sample size would roughly be identical.

To select participants, a stratified three-stage cluster proportional to the size sampling was used. The sample was stratified to provide adequate representation of both the rural and urban residences. An Excel spreadsheet was used to draw households from each selected cluster. One individual aged 25–64 years was randomly selected from each household using the Kish method.^11^

Data collection was conducted from 03 September 2013 to 24 October 2013 and household sociodemographic information was recorded *via* face-to-face interviews in the language spoken by the participant after BP and anthropometric measurements were collected.

### Variables of interest extracted from the STEPwise approach to surveillance (STEPS) survey database

The participants’ demographic variables included: gender, residence (rural or urban), age (in the range of: 25–64 years), marital status (grouped into: (1) married or cohabitating; (2) single), education level (grouped into: (1) no formal schooling; (2) primary school; and (3) secondary or higher) and occupation (grouped into: (1) public or private formal employment; (2) employment without or with uncertain income such as students, housekeepers or unemployed).

The STEPS questionnaire included the yes or no question on being ever screened for hypertension, and was: ‘Did a doctor or other health professional ever measure your BP?’.

Physical measurements: Anthropometric characteristics were weight (kg), height (m), body mass index (BMI; weight/height^2^, kg/m^2^) and waist circumference (WC, in cm). Height was measured to the nearest 0.1 cm using a stadiometer (SECA 214) on a subject without shoes, while weight was measured to the nearest 0.1 kg with a personal scale (SECA 813) on a lightly clothed subject without shoes. Waist circumference was measured to the nearest 0.1 cm (as per the WHO recommendations) with a measuring tape (SECA 203) at the midpoint between the last rib and the iliac crest, with the subjects standing upright and breathing normally. The BMI was used to characterise underweight (BMI < 18.5 kg/m^2^), normal (BMI = 18.5 kg/m^2^ – 24.9 kg/m^2^) overweight (BMI = 25 kg/m^2^ – 29.9 kg/m^2^) and global obesity (BMI ≥ 30 kg/m^2^) states and thus BMI ≥ 25 kg/m^2^ defined overweight/obesity.^12^ The WC was used to characterise abdominal obesity. Recently, the African Partnership for Chronic Disease Research (APCDR) specifically recommends the cut-offs of 81.2/81.0 cm for detecting abdominal obesity in men/women in SSA,^13^ while the cut-offs of 94/80 cm for men/women were previously recommended by the International Diabetes Federation (IDF).^14^

Systolic blood pressure (SBP, in mmHg) and diastolic blood pressure (DBP, in mmHg) values were measured three times using a mobile device (CardioChek™ 1708 PA, Indiana, United States [US]), with their mean value being used in the analysis. Individuals with mean values of SBP/DBP < 120/80 mmHg were considered normotensive, those with SBP between 120 mmHg and 139 mmHg and/or DBP between 80 mmHg and 89 mmHg were considered prehypertensive, while those with SBP/DBP ≥ 140/90 mmHg and/or medication as hypertensive.^15^

All devices for measurement were provided by the WHO and the measurements were carried out on the same day.

### Participants included in the analyses

After data collection, 105 individuals were not eligible or had invalid data regarding sex, and 10 had missing data on marital status or education level, 649 did not provide response to the yes/no question on ‘ever being or not screened for hypertension’, while 61 participants did not have valid data on BP and 144 on anthropometric parameters. In total, 3831 participants were included for the present secondary analyses. Without an increase of 20% assumed as the rate for possible nonresponse in the initial study design,^9^ the sample size was approximately 3828 participants.

### Statistical analyses

StataCorp™ Stata Statistical Software for Windows (Version 14.0, College Station, Texas, US) was used to analyse the data. The quantitative (continuous) variables are expressed as the means ± standard deviations (s.d.s), and the qualitative (categorical) variables are expressed as percentages (%) with 95% confidence interval (CI)s. Student’s *t*-test was used to compare quantitative variables, and the Chi-square test was used to compare categorical variables. We used both the APCDR and the IDF cut-offs to describe and compare abdominal obesity. Logistic regression analysis was performed using ‘ever been screened for hypertension’ (from the binary item, yes/no response) as the outcome, while the overweight/obesity state and abdominal obesity (according to the APCDR cut-offs) were explanatory variables, and all the six sociodemographic factors were the variables for adjustment. We proceeded by a progressive elimination of factors by decreasing the order of significance, i.e. with high level of the *p*-value. For all analyses, a *p*-value less than 5% was considered significant.

### Ethical considerations

The Ethics Committee for Health Research of the Ministry of Health of Burkina Faso approved the protocol of the WHO STEPS (deliberation No: 2012-12092; 05 December 2012). Each participant provided a written informed consent and the language used for the informed consent was the language spoken by the participant.

## Results

The sample was made up of 2009 (52.4%) female participants and the mean age was 38.5 ± 11.1 years. The participants were predominantly rural residents (78.8%), illiterates (75.9%) and married or cohabiting (85.8%) (**Table 1**).

**TABLE 1:** Sociodemographic characteristics of overall sample and for those who have ever been screened or never been screened for hypertension.

Variable	Overall (*N* = 3831)			Had never been screened for hypertension (*n* = 1592)			Previously screened for hypertension (*n* = 2239)			*p*-value
	*N*	%	CI	*n*	%	CI	*n*	%	CI	
**Residence**	-	-	-	-	-	-	-	-	-	0.0001
Rural area	3019	78.8	77.5–80.1	1386	45.9	44.1–47.7	1633	54.1	52.3–55.9	-
Urban area	812	21.2	19.9–22.5	206	25.4	22.4–28.5	606	74.6	71.5–77.6	-
**Sex**	-	-	-	-	-	-	-	-	-	0.0001
Male	1822	47.6	46.0–49.2	913	50.1	47.8–52.4	909	49.9	47.6–52.2	-
Female	2009	52.4	50.8–54.0	679	33.8	31.7–35.9	1330	66.2	64.1–68.3	-
**Age range (years)**	-	-	-	-	-	-	-	-	-	0.1700
25–34	1699	44.3	42.8–45.9	725	42.7	40.3–45.1	974	57.3	54.9–59.7	-
35–44	989	25.8	24.4-27.2	398	40.2	37.2–43.4	591	59.8	56.6–62.8	-
45–54	700	18.3	17.1–19.5	302	43.1	39.4–46.9	398	56.9	53.1–60.6	-
55–64	443	11.6	10.6–12.6	167	37.7	33.2–42.4	276	62.3	57.6–66.8	-
**Marital status**	-	-	-	-	-	-	-	-	-	0.4200
Married or cohabitating	3286	85.8	84.6–86.9	1357	41.3	39.6–43.0	1929	58.7	57.0–60.4	-
Single	545	14.2	13.1–15.4	235	43.1	38.9–47.4	310	56.9	52.6–61.1	-
**Occupation**	-	-	-	-	-	-	-	-	-	0.0001
Jobs without or regular income†	3598	93.9	93.1–94.7	1557	43.3	41.6–44.9	2041	56.7	41.6–44.9	-
Employees with formal and regular income‡	233	6.1	5.3–6.9	35	15.0	10.7–20.3	198	85.0	79.7–89.3	-
**Education level**	-	-	-	-	-	-	-	-	-	0.0001
No formal education	2907	75.9	74.5–77.2	1309	45.0	43.2–46.9	1598	55.0	53.1–56.8	-
Primary school	624	16.3	15.1–17.5	238	38.1	34.3–42.1	386	61.9	57.9–65.7	-
Secondary or more	300	9.8	7.0–8.7	45	15.0	11.2–19.6	255	85.0	80.4–88.8	-

CI, confidence interval at 95%.

† , Self-employed, house maker, jobless, students;

‡ , Workers with formal monthly salary in the public or private sectors.

Globally, 41.6% (95% CI: 40.0–43.1) of the participants had never been screened for hypertension before the 2013 STEPS survey when compared with those who had ever been screened, they had lower mean weight, WC and BMI, but were taller (**Table 2**). The proportion of participants with normal weight was high among those who had never been screened, while the proportion of those with overweight and obese status was lower. Using the IDF or APCDR cut-offs, there was a significantly lower prevalence of abdominal obesity among participants who had never been screened. The mean SBP was identical between the never and ever hypertension screening groups as well as the frequency of normal blood pressure or prehypertension. The prevalence of hypertension was significantly lower in the never-screened groups compared to their counterparts (17.3% *vs* 20.8%, *p* = 0.007).

**TABLE 2:** Comparison of the anthropometric and blood pressure features between adults who have ever been screened and who have never been screened for hypertension.

Variable	Overall (*N* = 3831)					Had never been screened for hypertension (*n*= 1592)					Previously screened for hypertension (*n* = 2239)					*p*-value
	*X̅*	s.d.	*N*	%	CI	*X̅*	s.d.	*n*	%	CI	*X̅*	s.d.	*n*	%	CI	
Height (cm)	166.2	8.4	9	-	-	166.6	8.2	-	-	-	165.9	8.5	-	-	-	0.0100
Weight (kg)	61.8	11.9	-	-	-	60.4	10.3	-	-	-	62.8	12.6	-	-	-	0.0001
Body mass index (kg/m^2^)	22.4	3.9	-	-	-	21.8	3.2	-	-	-	22.9	4.3	-	-	-	0.0001
Waist circumference (cm)	78.1	11.6	-	-	-	76.5	11.3	-	-	-	79.2	11.7	-	-	-	0.0001
Systolic blood pressure (mmHg)	123.3	17.4	-	-	-	123.2	16.8	-	-	-	123.4	17.8	-	-	-	0.7000
Diastolic blood pressure (mmHg)	78.4	10.9	-	-	-	77.5	10.4	-	-	-	79.0	11.3	-	-	-	0.0001
Differential blood pressure (mmHg)	45.0	12.0	-	-	-	45.8	11.9	-	-	-	44.4	12.1	-	-	-	0.0007
**Nutritional state**	-	-	-	-	-	-	-	-	-	-	-	-	-	-	-	0.0001*
Underweight	-	-	412	10.8	9.8–11.8	-	-	189	11.9	10.3–13.6	-	-	223.0	10.0	8.8–11.3	0.0600
Normal	-	-	2706	70.6	69.2–72.1	-	-	1196	75.1	72.9–77.2	-	-	1510 67.4		65.5–69.4	0.0001
Overweight	-	-	533	13.9	12.8–15.0	-	-	172	10.8	9.3–12.4	-	-	361.0	16.1	14.6–17.7	0.0001
Obese	-	-	180	4.7	4.1–5.4	-	-	35	2.2	1.5–3.0	-	-	145.0	6.5	5.5–7.6	0.0001
**Waist circumference categories (IDF cut-offs)**	-	-				-	-				-	-				0.0001
Absence of abdominal obesity	-	-	2975	77.7	76.3–79.0	-	-	1356	85.2	83.3–86.9	-	-	1619.0	72.3	70.4–74.2	-
Presence of abdominal obesity	-	-	856	22.3	21.0–23.7	-	-	236	14.8	13.1–16.7	-	-	620.0	27.7	25.8–30.0	-
**Waist circumference categories (SSA cut-offs)**	-	-	-	-	-	-	-	-	-	-	-	-	-	-	-	0.0001
Absence of abdominal obesity	-	-	2673	69.8	68.3–71.2	-	-	1240	77.9	75.8–79.9	-	-	1433.0	64.0	62.0–66.0	-
Presence of abdominal obesity	-	-	1158	30.2	28.8–31.7	-	-	352	22.1	20.1–24.2	-	-	806.0	36.0	34.0–38.0	-
**Blood pressure levels**	-	-	-	-	-	-	-	-	-	-	-	-	-	-	-	0.0290*
Normal	-	-	1543	40.3	38.7–41.8	-	-	654	41.1	38.7–43.5	-	-	889.0	39.7	37.7–41.8	0.3800
Prehypertension	-	-	1547	40.4	38.8–42.0	-	-	662	41.6	39.1–44.0	-	-	885.0	39.5	37.5–41.6	0.1900
Hypertension	-	-	741	19.3	18.1–20.6	-	-	276	17.3	15.5–19.3	-	-	465.0	20.8	19.1–22.5	0.0070

*X̅*, mean; s.d., standard deviation; CI, confident interval; SSA, sub-Saharan Africa; IDF, International Diabetes Federation.

* , Global *p*-value.

**Table 3** summarises the results of the logistic regression and shows that both the presence of overweight or obesity (adjusted odds ratio [aOR] = 1.3; 95% CI: 1.1–1.5; *p* = 0.03) and abdominal obesity (aOR = 1.3; 95% CI: 1.1–1.6; *p* = 0.002) were significantly associated with the uptake of hypertension screening (**Table 3**). In addition, favourable sociodemographic correlates were urban residence, female gender, older in age (in the range of 55–64 years), being educated, employed with formalised income and married or cohabiting.

**TABLE 3:** Associated factors with the uptake of the screening for hypertension.

Variable	Univariable analysis			Multivariable analysis		
	cOR	95% CI	*p*-value	aOR	95% CI	*p*-value
**Residence**	-	-	-	-	-	-
Rural area	1.0	-	-	1.0	-	-
Urban area	2.5	2.1–3.0	0.0001	1.5	1.3–1.9	0.0001
**Sex**	-	-	-	-	-	-
Male	1.0	-	-	1.0	-	-
Female	2.0	1.7–2.2	0.0001	2.2	1.9–2.5	0.0001
**Age range (years)**	-	-	-	-	-	-
25–34	1.0	-	-	1.0	-	-
35–44	1.1	0.9–1.3	0.2200	1.1	> 0.9–1.4	0.1200
45–54	> 0.9	0.8–1.2	0.8300	1.1	0.9–1.3	0.5700
55–64	1.2	> 0.9–1.5	0.0590	1.5	1.2–1.9	0.0001
**Marital status**	-	-	-	-	-	-
Single	1.0	-	-	1.0	-	-
Married or cohabitating	1.1	0.9–1.3	0.4200	1.4	1.2–1.7	0.0010
**Occupation**	-	-	-	-	-	-
Works without regular income†	1.0	-	-	1.0	-	-
Employees with regular income‡	4.3	3.0–6.2	0.0001	2.2	1.4–3.3	0.0001
**Education level**	-	-	-	-	-	-
No formal education	1.0	-	-	1.0	-	-
Primary school	1.3	1.1–1.6	0.0020	1.3	1.1–1.6	0.0090
Secondary or more	4.6	3.4–6.4	0.0001	3.0	2.0–4.5	0.0001
**BMI categories**	-	-	-	-	-	-
Normal	1.0	-	-	1.0	-	-
Underweight	0.9	0.8–1.2	0.5200	0.9	0.7–1.1	0.3300
Overweight or obesity	1.9	1.6–2.3	0.0001	1.2	1.1–1.5	0.0450
**Waist circumference**	-	-	-	-	-	-
Absence of abdominal obesity	1.0	-	-	1.0	-	-
Presence of abdominal obesity	2.0	1.7–2.3	0.0001	1.3	1.1–1.6	0.0020

CI, confident interval; cOR, crude odds-ratio; aOR, adjusted odds ratio; BMI, body mass index.

† , Self-employed, house maker, jobless or students;

‡ , Workers with formal monthly salary in the public or private sectors.

## Discussion

The adults who had never been screened for hypertension in Burkina Faso tended to have a slim appearance, supporting our hypothesis based on empirical observation, while the rates of prehypertension and hypertension were also important in these individuals.

### Unscreened adults in Burkina Faso

Proportion of Burkinabè adults who had never been screened for hypertension (41.6%) before this first national survey was substantial. The burden of undiagnosed hypertension in SSA is high and the screening is the STEPS survey leading to the diagnosis and treatment.^3^ Therefore, more appropriate strategies for screening are needed in Burkina Faso.

### Low anthropometric characteristics in participants who had never been screened

Body sizes were significantly correlated with body image perceptions and BP^16^ and therefore increased BMI was independently associated with a lower risk of being screened for pre-diabetes or diabetes^17^ (a metabolic disorder such as hypertension). For example, the term ‘diabesity’ has been coined to reflect the strong relationship between obesity and diabetes,^18^ yet in public health interventions or education, careless or unbalanced communication may have sometimes occurred. In some participants, the subjective misperception of being taller with lower weight, BMI and WC may have led to the belief that they were free from cardiovascular diseases. It is therefore necessary to be careful, especially in general communication, as metabolically obese with normal weight have been reported to reach up to 16% of apparently healthy individuals (especially women in the fourth quartile of normal weight) in Burkina Faso.^7^ Approximately 64% of Tanzanians and 43% of Nigerian women perceived an association between excess body weight and hypertension or cardiovascular disease^19,20^ and overweight/obesity was often associated with the occurrence of a cardiovascular event (stroke) in Beninese people.^21^

The frequent absence of both overweight or obesity and abdominal obesity in the participants who had never been screened may potentially lead to the high degree of body image satisfaction in health domains^22^ and limit their engagement in screening. The finding that the presence of both overweight or obesity and abdominal obesity was associated with screening uptake in a multivariable analysis reinforces our view that weight gained, by altering body image perceptions, is a potential driver of screening uptake. Perhaps the popularised and disseminated public health message or the invitation to be screened for hypertension was unclear or more selective, targeting only overweight/obese individuals or those who subjectively considered themselves to be overweight or obesity. Thus, there is a need for incitement messages for all adults, regardless of their perceptions of body size. However, more research, including qualitative studies, is needed to accurately assess the effect of self-perceived body size or image on misconceptions and adherence^23^ to screening. The results should help to specify public health messages targeting misconceptions of ‘apparently healthy body size’ as a potential barrier to hypertension screening, especially as metabolically obese with normal weight is substantial in some of the groups.^7^

### Blood pressure characteristics among participants who had ever or never been screened

The mean SBP was identical between the two groups and there was a similar prevalence of normotensive or prehypertensive individuals. The 11-year risk factor of hypertension in prehypertensive Mauritians^24^ and the high prevalence of prehypertension (even in the never screened groups) should be considered a major public health concern in Burkina Faso, where the population is predominantly young (mean 38.5 ± 11.1 years in our study). In those who had never been screened, the rate of progression to hypertension may not be detected and was slow. Although the prevalence of hypertension (17%) was statistically lower among participants who had never been screened, it is a significant public concern as they may benefit from early antihypertensive therapy to reduce the burden of potential complications. In particular, people who had never been screened could not comply with healthy lifestyle behaviours such as the practice of physical activity, consumption of fruit and vegetables, abstinence from alcohol and/or tobacco use, salt consumption, among others. It can be hypothesised that this level of unknown hypertension may contribute to the high number of surprising cardiocerebrovascular events recorded in the Burkina Faso hospital settings (hypertension was reported in 82% of stroke patients).^25^

### Favourable sociodemographic factors for hypertension screening

Most of the sociodemographic parameters were associated with hypertension screening uptake and were consistent with the common sociodemographic or socioeconomic correlates of inequalities in undiagnosed hypertension in LMICs.^26^ Urban participants may benefit from geographical proximity to healthcare facilities, improved education about hypertension and regular invitations to screening.^27^ Employees may be frequent screeners because of their high-income levels. Education leads to a better understanding of hypertension issues. Even attending the first level of the national education system was helpful, so health literacy should be implemented with a focus on cardiovascular health education and adapted for the general population.^28^ Female gender was more affected by overweight or obesity, especially in urban areas,^29^ leading to the screening. This attitude should place women in the key role of the family-based approaches to cardiovascular health promotion or risk reduction.^30^ This is appropriate, as being in the married or cohabiting groups was also associated with the practice of hypertension screening. Older adults (55–64 years) may benefit from their general life experience. In fact, the average age of patients with cardiocerebrovascular events admitted to country hospitals was reported to be around 60 years,^31^ and hypertension was found in most of the cases.^25^ People in their 60s may know others (parents, siblings, colleagues, etc.) who have had such events (stroke, ischemic heart disease, etc.). Knowing about such events makes them want to take precautions and get screened.

### Insights from our findings

Metabolically obese normal weight (cardiometabolic disorders in those with normal BMI) could occur in up to 16% of some apparently healthy individuals^7^ and in the Burkina Faso referral hospitals, hypertension was reported in 82% of stroke patients.^25^ The mean BMI of those who had never been screened was not significantly different from the mean BMI of stroke patients admitted to these hospitals (22.1 ± 4.8 kg/m^2^).^31^ Motivating thin-looking people to be screened for hypertension is an important issue, and they should be made aware of their potential vulnerability through specific, tailored recommendations, without triggering a reaction of panic, stress or social stigmatisation. Community-based interventions to improve mother–child^32^ care and prevent specific infectious diseases^33^ have been successfully tested in Burkina Faso. This framework of interventions can be tailored for hypertension screening and management. Therefore, interventions at the primary care level supported by specific community-based interventions will be useful.

As a follow-up to our findings, the hypothesis that thin people are less likely to be screened for hypertension is currently being tested in urban population in Burkina Faso using a mixed method. Data have been collected and quantitative analyses are ongoing, while a further qualitative study will use individual and group interviews with a still limited sub-sample.

### Limitations

The STEPS method was interesting in the hypothetical case where the subject would be informed of their hypertensive status by a health worker or doctor; however, it could happen that individuals themselves could detect their hypertensive status by using automatic electronic devices. The analysis did not compare levels of unhealthy lifestyle behaviours between the two groups. Furthermore, this study was not doubled with a qualitative study which could explore how the anthropometric sizes influence the self-perceived body image dissatisfaction or satisfaction in relation to the attitudes and practices towards hypertension screening.

## Conclusion

Although sociodemographic characteristics were important in analysing factors associated with uptake of hypertension screening among Burkinabè adults, our study suggests that the effects of self-perceived body size should also be considered. Thinness may be a potential barrier to the adherence to hypertension screening, and this was consistent with empirical observations in Burkina Faso. Further research, including qualitative studies, should provide sufficient evidence to support this hypothesis, which in turn will help to refine public health messages and interventions to increase screening uptake among all adults in the country. As has been undertaken successfully to improve mother–child care and prevent specific infectious diseases, an adapted framework of interventions at the primary care and community levels should be tailored for hypertension screening and management.
